# Microbiome Research as an Effective Driver of Success Stories in Agrifood Systems – A Selection of Case Studies

**DOI:** 10.3389/fmicb.2022.834622

**Published:** 2022-07-04

**Authors:** Rocío Olmo, Stefanie Urimare Wetzels, Jaderson Silveira Leite Armanhi, Paulo Arruda, Gabriele Berg, Tomislav Cernava, Paul D. Cotter, Solon Cordeiro Araujo, Rafael Soares Correa de Souza, Ilario Ferrocino, Jens C. Frisvad, Marina Georgalaki, Hanne Helene Hansen, Maria Kazou, George Seghal Kiran, Tanja Kostic, Susanne Krauss-Etschmann, Aicha Kriaa, Lene Lange, Emmanuelle Maguin, Birgit Mitter, Mette Olaf Nielsen, Marta Olivares, Narciso Martín Quijada, Marina Romaní-Pérez, Yolanda Sanz, Michael Schloter, Philippe Schmitt-Kopplin, Sarah Craven Seaton, Joseph Selvin, Angela Sessitsch, Mengcen Wang, Benjamin Zwirzitz, Evelyne Selberherr, Martin Wagner

**Affiliations:** ^1^FFoQSI GmbH - Austrian Competence Centre for Feed and Food Quality, Safety and Innovation, Tulln, Austria; ^2^Unit of Food Microbiology, Institute of Food Safety, Food Technology and Veterinary Public Health, University of Veterinary Medicine, Vienna, Austria; ^3^Symbiomics Microbiome Solutions, Florianópolis, Brazil; ^4^Genomics for Climate Change Research Center, Universidade Estadual de Campinas, Campinas, Brazil; ^5^Centro de Biologia Molecular e Engenharia Genética, Universidade Estadual de Campinas, Campinas, Brazil; ^6^Departamento de Genética e Evolução, Instituto de Biologia, Universidade Estadual de Campinas, Campinas, Brazil; ^7^Institute of Environmental Biotechnology, Graz University of Technology, Graz, Austria; ^8^Leibniz Institute for Agricultural Engineering and Bioeconomy (ATB), Potsdam, Germany; ^9^Institute for Biochemistry and Biology, University of Potsdam, Potsdam, Germany; ^10^Food Bioscience, Teagasc Food Research Centre Moorepark, Fermoy, Ireland; ^11^APC Microbiome Ireland and VistaMilk, Cork, Ireland; ^12^SCA, Consultoria em Microbiologia Agrícola, Campinas, Brazil; ^13^Brazil National Association of Inoculant Producers and Importers (ANPII), Campinas, Brazil; ^14^Department of Agricultural, Forest and Food Science, University of Torino, Torino, Italy; ^15^Department of Biotechnology and Bioengineering, Technical University of Denmark, Kongens Lyngby, Denmark; ^16^Laboratory of Dairy Research, Department of Food Science and Human Nutrition, Agricultural University of Athens, Athens, Greece; ^17^Department of Veterinary and Animal Sciences, University of Copenhagen, Frederiksberg, Denmark; ^18^School of Life Sciences, Pondicherry University, Puducherry, India; ^19^Bioresources Unit, Center for Health & Bioresources, AIT Austrian Institute of Technology GmbH, Tulln, Austria; ^20^Research Center Borstel, Leibniz Lung Center, Airway Research Center North (ARCN), German Center for Lung Research (DZL), Borstel, Germany; ^21^Institute for Experimental Medicine, Christian Albrechts University, Kiel, Germany; ^22^Microbiota Interaction With Human and Animal Team (MIHA), Micalis Institute, Université Paris-Saclay, INRAE, AgroParisTech, Jouy-en-Josas, France; ^23^BioEconomy, Research & Advisory, Copenhagen, Denmark; ^24^Department of Animal Science, Faculty of Technical Sciences, Aarhus University, Tjele, Denmark; ^25^Microbial Ecology, Nutrition and Health Research Unit, Institute of Agrochemistry and Food Technology, Spanish National Research Council (IATA-CSIC), Valencia, Spain; ^26^Research Unit Comparative Microbiome Analysis, Helmholtz Center Munich, Neuherberg, Germany; ^27^Research Unit Analytical Biogeochemistry, Helmholtz Center Munich, Neuherberg, Germany; ^28^Indigo Agriculture, Charlestown, MA, United States; ^29^State Key Laboratory of Rice Biology & Ministry of Agricultural and Rural Affairs Laboratory of Molecular Biology of Crop Pathogens and Insects, Institute of Pesticide and Environmental Toxicology, Zhejiang University, Hangzhou, China; ^30^Institute of Food Science, University of Natural Resources and Life Sciences, Vienna, Austria

**Keywords:** microbiome-based applications, agrifood system, multi-omics analyses, success case studies, food microbiome

## Abstract

Increasing knowledge of the microbiome has led to significant advancements in the agrifood system. Case studies based on microbiome applications have been reported worldwide and, in this review, we have selected 14 success stories that showcase the importance of microbiome research in advancing the agrifood system. The selected case studies describe products, methodologies, applications, tools, and processes that created an economic and societal impact. Additionally, they cover a broad range of fields within the agrifood chain: the management of diseases and putative pathogens; the use of microorganism as soil fertilizers and plant strengtheners; the investigation of the microbial dynamics occurring during food fermentation; the presence of microorganisms and/or genes associated with hazards for animal and human health (e.g., mycotoxins, spoilage agents, or pathogens) in feeds, foods, and their processing environments; applications to improve HACCP systems; and the identification of novel probiotics and prebiotics to improve the animal gut microbiome or to prevent chronic non-communicable diseases in humans (e.g., obesity complications). The microbiomes of soil, plants, and animals are pivotal for ensuring human and environmental health and this review highlights the impact that microbiome applications have with this regard.

## Introduction

Microbiome research has been intensifying in recent years in terms of the numbers of publications, research programs and complexity. A query performed using the PubMed repository (visited on 4 April 2022)^1^ revealed that in the last 10 years, the number of publications including the term “microbiome” in the title and/or the abstract was 40,565, where 24,261 of them (61%) were published just in the last 3 years. The term “microbiome” was first described by Whipps et al. (1988) and has been recently redefined by Berg et al. (2020) as microbial communities (including prokaryotes, fungi, protozoa, and other micro-eukaryotes) that occupy well-defined habitats and their “theatre of activity.” This “theatre of activity” includes microbial structures, metabolites/signal molecules, and mobile genetic elements, including transposons, phages, viruses, and relic DNA, embedded in the environmental conditions of the habitat (Berg et al., 2020). The enormous potential of advanced sequencing technologies have led to a rapid evolution in microbiome research in the last decades, which has become a topic of great interest in science, society, and industry. Innovation actions address significant challenges within microbiome applications for sustainable food systems, yielding outputs of commercial relevance across many product and process areas. The European-funded coordination and support action MicrobiomeSupport^2^ acts by setting a basis for a common R&D framework for food system microbiomes. To achieve this, MicrobiomeSupport conducted mapping exercises (Meisner et al., 2022), proposed common terminology and standards (Berg et al., 2020; Ryan et al., 2021) and is developing recommendations for a strategic research and innovation agenda. Microbiomes are an integral component of many scientific fields related to the agrifood system, such as medical, nutrition, feed and food, veterinary, and environmental sciences. The mapping exercise showed that most publications and research projects address human microbiomes and, more precisely, gut microbiomes. The second biggest thematic cluster comprises publications on soil and plant microorganisms, and research projects on environmental (mostly soil) and plant microbiomes and also projects on primary production systems (mostly agriculture). Within the research projects targeting microbiomes in food products and processing environments, microbiomes used as additives or health supplements and microbiomes of fermented foods (such as starters or ripening cultures) are the most investigated fields (Meisner et al., 2022).

In the last years, the application of high-throughput DNA sequencing (HTS) approaches has revolutionized the way microbes are investigated. Different sequencing platforms are available and their constant improvement allows the generation of billions of base pairs of data in a reasonable time and price (Quijada et al., 2020b). The success of the different HTS approaches relies on their capability to define, with a high level of depth and accuracy, the microbial communities occurring in a specific environment, as well as their potential activities and functions (Cocolin and Ercolini, 2015; Gerner-Smidt et al., 2019). These techniques have been successfully applied to several areas from “Farm-to-Fork,” such as the investigation of microbial dynamics along the food chain and during food fermentation, the screening of microorganisms and/or genes that are associated with hazards for animal and human health in feeds, foods, and processing environment, or the identification of novel potential probiotics or metabolites. To exploit the rapid growth of research and innovation in the field of agrifood microbiomes, it has to be ensured that this knowledge is integrated into industry and that legal and regulatory considerations are made. Many microbiome-based applications exist nowadays and the aim of this review is to expose selected cases where the increase in microbiome knowledge has led to advancement within the “Farm-to-Fork” context. In the following sections, we will elaborate on 14 selected microbiome success stories to demonstrate the crucial role of microbiome research associated with the agrifood system. Among the microbiome success stories available in the literature, we decided to focus on 14 examples taken from partners of the MicrobiomeSupport consortium that have demonstrated significant impacts on plant, animal, human, and environmental health. These case studies focus on the microbiome role and range from production of plant raw materials for food, feed supplement products, production and preservation of food, health benefiting effects from food.

## Example Success Stories

In this collection of case studies, we provide an overview of agrifood success stories based on microbiome research that meet one or more of the following criteria: (i) Microbiome-based innovations in the agrifood chain context that led to commercialization, (ii) Microbiome-based mitigation strategies implemented by the industry to increase product yield and quality or decrease economic losses during production, (iii) Microbiome-based innovations in the agrifood sector that helped improve plant, animal, and human health, (iv) Microbiome-based knowledge that led to improved food safety management systems, or (v) Amendments of legal regulations based on microbiome research. For the purpose of this review, the MicrobiomeSupport consortium members have selected 14 example case studies matching at least one of the above-mentioned criteria from different sectors, including plant health, feed products and livestock health, food production and human health (Table 1).

**Table 1:** Case studies addressed in the manuscript organized into different sectors and their main findings and references.

Sector		Case study	Main finding	References
Plant health	1	Disease resistance conferred by the plant seed microbiome.	*Sphingomonas melonis* ZJ26, which naturally occurs as an endophyte in rice seeds, was shown to shape the host phenotype by evoking disease resistance against the emerging plant pathogen *Burkholderia plantarii*.	Matsumoto et al., 2021
	2	Boosting sustainable crop productivity through nitrogen-fixing microorganisms: the Brazilian case.	Diazotrophic bacteria supply N to the plants through biological nitrogen fixation (BNF). These microbes have been applied to partially or completely replace chemical N fertilizers in agricultural systems. The potential of BNF for more sustainable food production is demonstrated in Brazilian soybean plantation.	Hungria et al., 2015; Rondina et al., 2020; Santos et al., 2021
	3	Fungal-based BioAg products for improved plant growth.	A seed-borne inoculum of *Penicillium bilaiae* (filamentous fungus) is used to increase the availability and accessibility of soil phosphorus to plant roots through the colonization of the root system and production of organic acid exudates, strengthen seedling vigor and hereby improving growth performance.	Gómez-Muñoz et al., 2018
	4	*Bacillus* sp. S4—from lab to the field.	A *Bacillus simplex* (endophytic bacterium) strain isolated in the frame of fundamental research has led to a commercially available product for the improvement of drought-stress tolerance in maize.	Naveed et al., 2014; Mitter et al., 2016
Feed products and livestock health	5	Industry develops new type of gut health feed additives, microbially processed.	Co-fermented rapeseed meal and seaweed enhanced colon mucosal development and reduced signs of intestinal inflammation. Piglet performance, intestinal development and health indicators were improved when in-feed zinc oxide was replaced by this feed additive.	Satessa et al., 2020a,b
	6	Improved animal gut microbiome by new feed additives, for lowering use of antibiotics.	Xylooligosaccharides prebiotics improved porcine gut health. Furthermore, such prebiotic feed additives could be produced affordable and in large scale, as a side-stream to Green Biorefinery.	Dotsenko et al., 2018
	7	Probiotics in poultry feed.	Microbiome modulation with the help of probiotics have become a promising biological strategy to tackle infections and intoxications in poultry animals.	Liew et al., 2019; Chang et al., 2020
Food production	8	Multi-omics approach reveals the importance of the use of autochthonous microbiome in precision meat fermentation.	Autochthonous microbiome display an extensive pool of genes with adapted metabolic functions, which can be potentially used as starter culture to prevent the loss of typicity and guarantee quality and safety.	Ferrocino et al., 2018; Franciosa et al., 2021
	9	The microbiota of home-made and industrial kefir produced in Greece.	The microbiota of home-made and industrial kefir samples produced in Greece was elucidated using both culture-based and amplicon-based sequencing analyses. Bacteria and yeast strains belonging to species with technological importance was isolated and identified and the three microbial ecosystems, i.e., home-made grains, home-made drinks, and industrial drinks, was differentiated.	Kazou et al., 2021
	10	Investigating the microbial basis for the pink discoloration defect in cheese.	Identification of *Thermus* as a contributor to the cheese pinking defect by employing HTS-based approaches. The development of a qPCR-based assay to identify the route *via* which these microbes entered the food chain.	Quigley et al., 2016; Yeluri Jonnala et al., 2021
	11	Sources and transmission routes of microbial populations throughout a meat processing facility.	The microbiome composition and distribution was shown in a pork-processing plant and hints for increased food safety assessments and better hygiene standards could be provided.	Zwirzitz et al., 2020
Human health	12	Identification of D-tryptophan as a microbiome modulating prebiotic compound with the potential to mitigate asthma.	The identification of a metabolite from a probiotic bacterium derived from fermented food, which modulate the microbiota in the human gut and reduce symptoms of asthma in a murine model.	Kepert et al., 2017
	13	A multi-fiber enriched bread to feed the gut microbiota.	Science-based selection of 7-fibers to promote diverse ecological niches in the gut microbiota, improve cardiometabolic profiles and further prevent cardiometabolic risk.	Ranaivo et al., 2022
	14	*Bifidobacterium pseudocatenulatum* CECT 7765 for preventing metabolic complications associated with obesity in humans.	These microbiome studies led to discover the potential of the strain *B. pseudocatenulatum* CECT 7765, isolated from a healthy breast-fed baby, to reduce the inflammation associated with obesity and by doing so minimizing the risk of metabolic complications more effectively than dietary counseling alone.	Cano et al., 2013; Moya-Pérez et al., 2014, 2015; Benítez-Páez et al., 2016; Sanchis-Chordà et al., 2019

### Plant Health Sector

One of the greatest challenges for agriculture in the upcoming decades is to meet the demand for food production caused by a growing human population, which is expected to reach 10 billion in 2050 (Tilman et al., 2011; Ray et al., 2013). Food production will need to increase by roughly 70% by 2050 and food producers will need to simultaneously limit their environmental impact (Crist et al., 2017). Therefore, the development of environmentally sustainable agriculture is necessary, where biofertilizers, biostimulants, and biopesticides will play a fundamental role. The studies performed on plant and soil microbiomes during the last decades have highlighted the dynamic and complex interactions that occur between microorganisms, plants and soil (Hartmann et al., 2009; Vorholt, 2012; Berg et al., 2014a, 2016; Hardoim et al., 2015; Hassani et al., 2018). Studies on topics such as plant tolerance and resistance to diseases, and/or abiotic stresses and nutritional, mineral and vitamin supplementation, have led to the implementation of microbial-based products, which are now successfully registered as plant growth-promoting (PGP) agents in several countries and available on the market (Calvo et al., 2014; Wallenstein, 2017; Woo and Pepe, 2018). In recent years, a growing number of studies have suggested that plant-microbe interactions promote plant health and development to a larger degree than previously acknowledged (Turner et al., 2013; Berg et al., 2014b). Complex communities of archaea, bacteria, and fungi can live as epiphytes or endophytes in different host tissues (Turner et al., 2013) and have been considered as an extension of the host’s functional repertoire by providing a second genome crucial for the plant health and growth (Berendsen et al., 2012; Lemanceau et al., 2017). In this context, soil plays a crucial role as a source of additional microorganisms and the transmission of plant associated microbiota from one plant generation to the next one (Sánchez-Cañizares et al., 2017).

### PGP Candidates for Plant Disease-Prevention

Plant pathogens are causing substantial losses in the production of plant-based food products globally. Crop protection against pests and diseases has been mainly achieved by pesticides that are harmful to the environment and non-target organisms. However, an increased number of recent studies targeting the seed microbiome have demonstrated their ability to improve crop sustainability and production without the need of chemical substances (Berg and Raaijmakers, 2018). Different microbiome studies have indicated that up to 20,000 microbial species and up to 2 billion of bacterial cells can be present in one single seed (Adam et al., 2016; Johnston-Monje et al., 2016; Shade et al., 2017). The seed microbiota of plants not only includes bacteria, but also fungi as well as archaea (Wassermann et al., 2019; Taffner et al., 2020). Seed-derived bacteria were found to play a crucial role in the assembly of tissue-specific microbial communities in plant seedlings (Abdelfattah et al., 2021). When the establishment of seed endophytes was investigated, it was shown that endophytic communities can be shaped by targeted introduction of microbial inocula (Rezki et al., 2016). Mitter et al. (2017) showed for the first time that bacteria can be selectively introduced into maize seeds. Other studies provided evidence that plant breeding resulted in the divergence of the seed microbiome in various crop plant species (Adam et al., 2016; Abdullaeva et al., 2020; Chen et al., 2020). In targeted approaches, where different generations of the same plant cultivar were compared in terms of their seed microbiome composition and structure, it was shown that bacterial endophytes can substantially differ between generations. The same studies also showed that while the seed microbiome is subjected to certain dynamics, it could still maintain beneficial bacteria (Bergna et al., 2018; Matsumoto et al., 2021).

**Figure 1:**
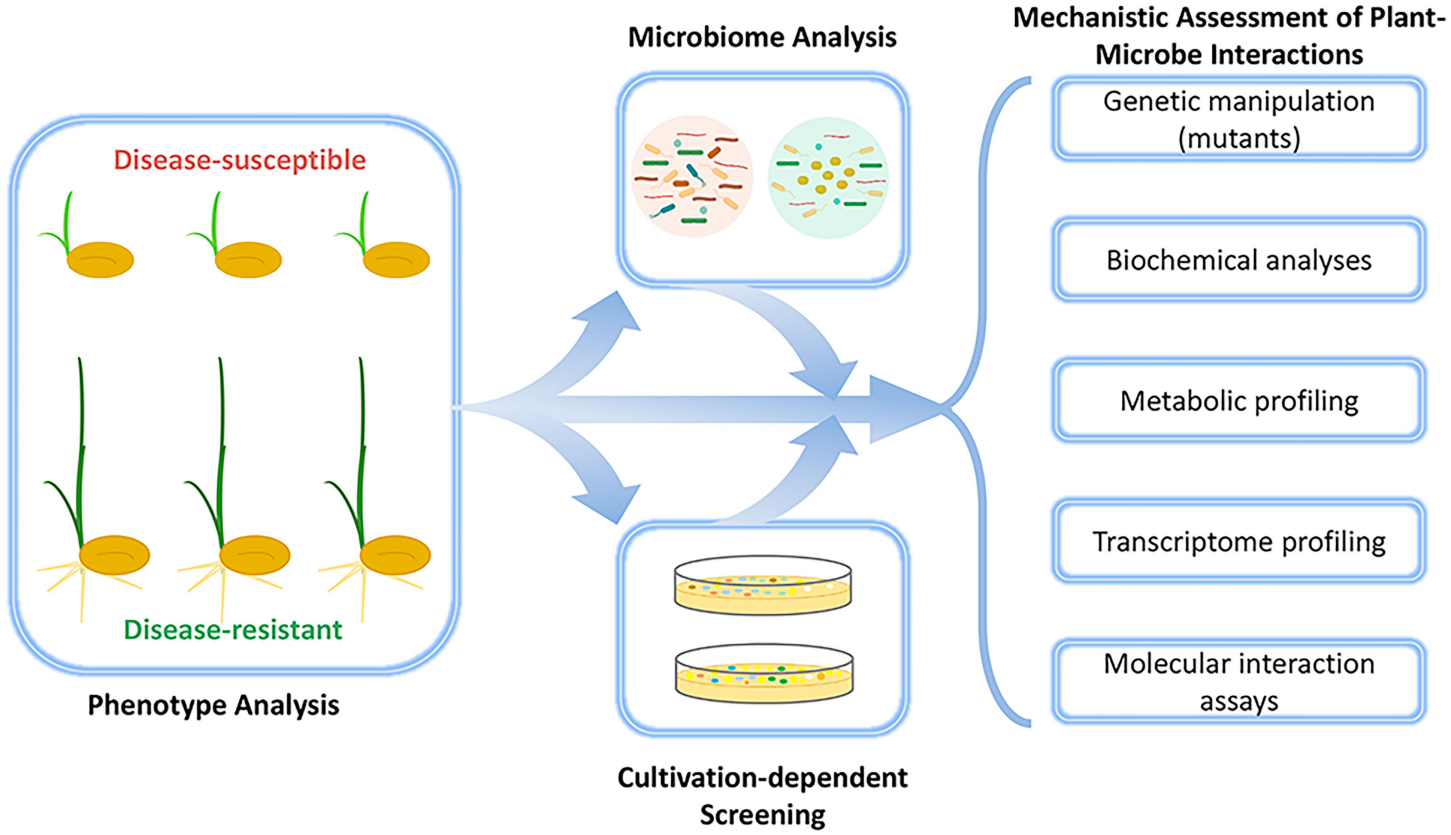
Schematic workflow of the integrative approach that led to the discovery of the first seed-endophytic bacterium that confers holistic disease resistance to rice plants. Its discovery was facilitated by large-scale microbiome analyses that were complemented with a series of cultivation-dependent and-independent experiments. The approach led to the discovery of *Sphingomonas melonis* ZJ26 that shapes a disease-resistant rice phenotype.

In the first success story presented here (case study 1; Table 1), we highlight the discovery of the first seed-endophytic bacterium with holistically disease-preventing traits which was facilitated by integrative approaches that included not only conventional microbiome analyses, but also large-scale disease occurrence monitoring (Matsumoto et al., 2021). First assessments indicated that diverse members of the bacterial genus *Sphingomonas* were accumulated and transmitted across successive generations in disease-resistant rice seeds and conferred resistance to disease-susceptible phenotypes against the plant pathogen *Burkholderia plantarii*. Using culture-based approaches, Matsumoto et al. (2021) confirmed an endophytic *Sphingomonas melonis* strain (ZJ26) as the core constituent of the seed-endophytic bacterial community in disease-resistant phenotypes. More profoundly, *S. melonis* ZJ26 restored the phenotypic plasticity in disease-susceptible rice seeds upon exposure to *B. plantarii*. The identification of *S. melonis* ZJ26 as the resistance-conferring agent by the integration of HTS data, gene mutagenesis, and molecular interaction assays facilitated the discovery of its principal mode of action that underlies pathogen inhibition by *S. melonis* ZJ26 (Figure 1), which is based on the disruption of the pathogen’s virulence signaling cascade by anthranilic acid secretion from *S. melonis* ZJ26 (Matsumoto et al., 2021). The strategy employed here demonstrates that microbiome studies that integrate multiple approaches and data types can provide much more mechanistic insight than those that rely on HTS alone. Although this discovery is unique so far, one can assume that a similar approach will facilitate the discovery of microorganisms with analogous functions in other plant species and pave the way for the implementation of disease-preventing microorganisms in sustainable agriculture.

### Enabling Sustainable Agriculture With Microbe-Based Products

Crop production is often limited by the availability of essential macro- and micronutrients. Nitrogen (N) is one of the most limiting nutrients affecting crop yield, and thus applied in large quantities worldwide (Zhang X. et al., 2015). However, excessive application and continued dependence on N fertilizers raise the cost of crop production and increases environmental degradation. Urea, the most used N fertilizer, is manufactured by an energy-intensive process that requires fossil fuels and generates large amounts of greenhouse gases (GHG; Woods et al., 2010). Microbial inoculants composed of atmospheric nitrogen-(N_2_)-fixing bacteria (diazotrophs) have been effectively shown to partially or completely replace chemical N fertilizers in agricultural systems (Hungria et al., 2015; Santos et al., 2019, 2021; Rondina et al., 2020). Diazotrophic bacteria supply plants with N through biological nitrogen fixation (BNF), a process that involves the nitrogenase, an enzyme that catalyzes the conversion of atmospheric N_2_ into ammonia (NH_3_), which can be easily assimilated by plants (Kuypers et al., 2018). Diazotrophs include free-living soil bacteria (e.g., *Azotobacter* spp.), associative bacteria (e.g., *Azospirillum* spp.), and symbiotic bacteria (e.g., *Bradyrhizobium* spp.; Kaschuk and Hungria, 2017). In legumes, such as soybeans, symbiotic associations are established by the formation of root nodules, which are specialized root structures that harbor N_2_-fixing microbes. Within the nodules, plants provide carbon sources and other nutrients to the bacteria, while the bacteria deliver excess fixed N_2_ to the plant (Saha et al., 2017).

**Figure 2:**
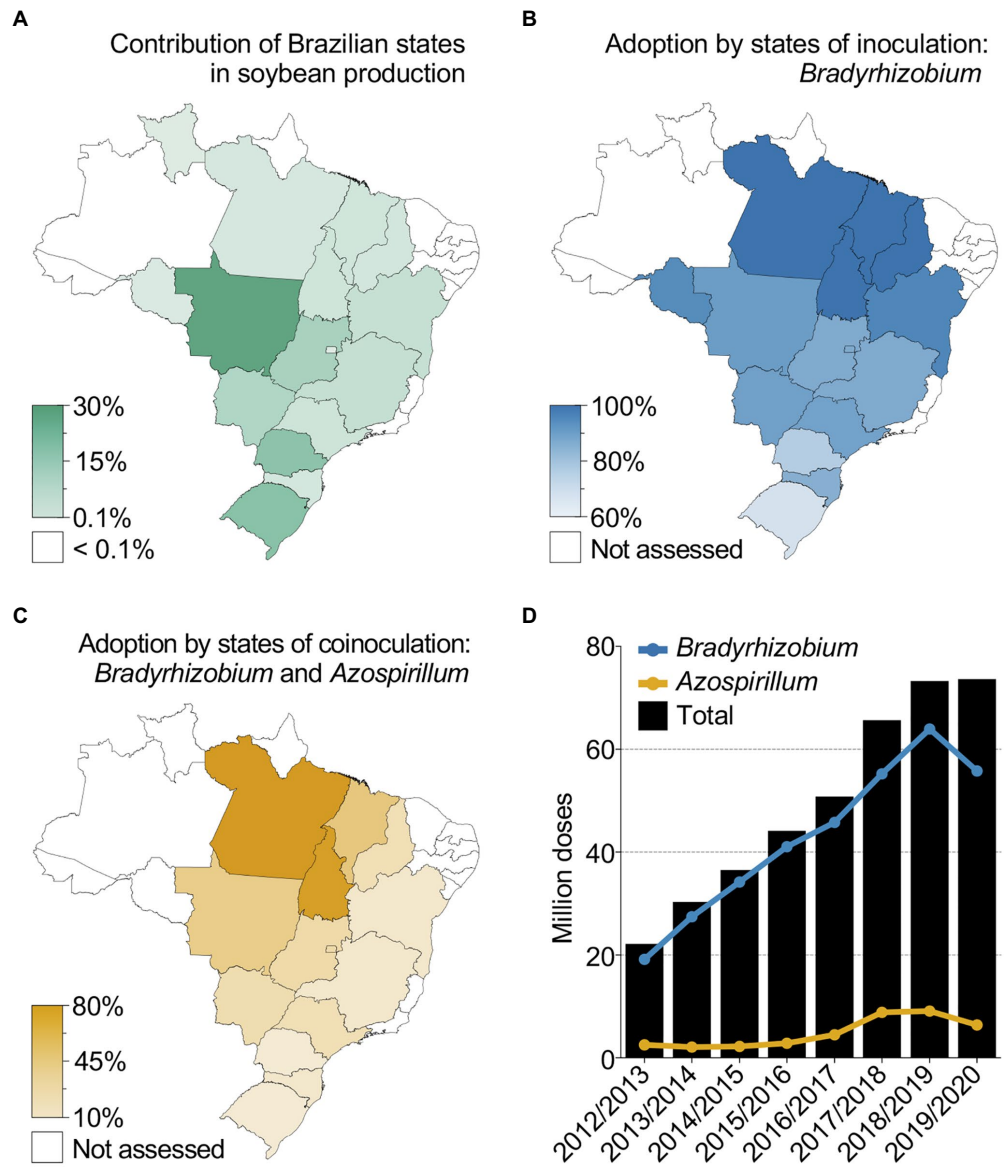
Soybean microbial inoculation and co-inoculation in Brazil. **(A–C)** Individual state contributions to soybean production **(A)**, adoption by states (% of area) of *Bradyrhizobium*-based inoculants **(B)** and adoption by states (% of area) of *Azospirillum*-based inoculants **(C)** for co-inoculation in Brazil in the 2019/2020 soybean crop season. **(D)** Total number of inoculant doses for BNF, *Bradyrhizobium*-based inoculant applied to soybean and *Azospirillum*-based inoculant for grasses, sold in Brazil over several years. A partial number of doses are shown in the 2019/2020 crop season due to incomplete data collection (Source: ANPII/Spark). Data from the 2019/2020 crop season were based on 3,551 interviewed farmers covering an extrapolated soybean planted area of 98% with a 95% confidence level and a 1.6% margin of error.

Case study 2 (Table 1) illustrates the successful use of N-fixing microbes to boost sustainable crop productivity in Brazil (Hungria et al., 2015; Rondina et al., 2020; Santos et al., 2021). In the 2019/2020 crop season, 37 million hectares of cultivated soybean in Brazil produced an average yield of 3.4 tons ha^−1^ (Figure 2A). Considering that soybean requires ~80 kg of N per ton of grain and that the use efficiency of N fertilizers rarely exceeds 50% due to leaching, volatilization and denitrification processes (Hungria et al., 2007; Lassaletta et al., 2014; Zhang X. et al., 2015), a total of ~20 million tons of N, equivalent to ~43 million tons of urea, would be necessary to sustain Brazilian soybean plantations without BNF. Additionally, the replacement of chemical fertilizers by N_2_-fixing microbes contribute substantially to the reduction of GHG emissions. Assuming that each kilogram of N fertilizer corresponds to nearly 10 kg of CO_2_-equivalent GHG emissions (Brock et al., 2012), approximately 430 million tons of CO_2_-equivalent gases would be released annually if no N_2_-fixing microbes were used in Brazilian soybean plantations. However, because Brazil developed the most successful program for using *Bradyrhizobium*-containing inoculants, there was a considerable reduction in environmental degradation and production costs. More recently, the adoption of inoculants containing N_2_-fixing microbes for soybean reached 79% (Figure 2B; Santos et al., 2021), which represents approximately US$ 10.2 billion in saved N fertilizers. The robust science and development efforts behind the *Bradyrhizobium* BNF program have also encouraged the development of inoculants containing biostimulants with N_2_-fixing bacteria for grasses. The first inoculant for grasses based on *Azospirillum brasilense* was registered in 2009 in Brazil, initially designed for application with corn, wheat, and rice. In recent years, several studies and field data have pointed out the benefit of co-inoculating *Bradyrhizobium* spp. and *A. brasilense* strains in legumes (Hungria et al., 2013, 2015; Chibeba et al., 2015; Santos et al., 2021). From 2017 to 2019 co-inoculation of soybean with both species has been adopted by farmers and reached 25% of the total soybean planted area in Brazil (Figure 2C; Santos et al., 2021). In total, the number of inoculant doses reached more than 73 million in the 2019/2020 crop season (most farmers use more than one dose per hectare), with an significant increase in *A. brasilense*-based inoculants in the last 4 years, reflecting a growing practice of co-inoculation in soybean (Figure 2D; Santos et al., 2021). It took almost 40 years of intermittent use of soybean inoculants in Brazil to develop and implement regulation and mechanisms that eventually culminated in the adoption of inoculants in the country.

Plant accessibility (through their root system) to sufficient phosphorus (P) in soil is a well-known and widespread limiting factor for the establishment and growth of crop plants (Lambers et al., 2006). Phosphate-solubilizing microorganisms are reported to solubilize otherwise unavailable inorganic P in the soil (Richardson and Simpson, 2011). Considering this, a new biological method for providing additional plant-accessible P has been developed to support the seedling stage, therefore reducing the use of P fertilizers. It builds on knowledge of certain filamentous fungi (*Penicillium* genus) that secrete organic acids in concentrations required for dissolving inorganic P in the soil (Dutton and Evans, 1996). Therefore, a seed-borne inoculum of these fungi can be used to modify and enrich the rhizosphere microbiome in both organismal composition and function (Gómez-Muñoz et al., 2018; Raymond et al., 2021). This is the basis of case study 3 (Table 1), which shows how the inoculation of a species of *Penicillium* onto plant seeds can strengthen seedling vigor, thereby improving growth performance and tolerance to pests and disease (Gómez-Muñoz et al., 2018; Raymond et al., 2021). This innovation led to a commercially available PGP product. For this to be realized, it was important to understand the biology of fungal species within the genus *Penicillium*. They are fast growing, strong colonizers, with a rich secreted metabolome, often including antimicrobial metabolites, which provides them with a competitive advantage in the environment (on the root surface and in the rhizosphere). Furthermore, the vulnerable phase (from inoculum to active growth in the rhizosphere) can be overcome by the rapid germination of a high percentage of the conidia typical of *Penicillium* spp. The species found to perform best for this type of application by the biotech industry was *Penicillium bilaiae*, which does not produce metabolites toxic to humans or other animals. Notably, the first reported producer of oxalic acid in *Penicillium*, *Penicillium oxalicum* (Currie and Thom, 1915) produces mycotoxins and can be pathogenic (Kubátová et al., 2019), but has nonetheless been suggested as a soil fertilizer (Singh and Reddy, 2011). Similarly, another cereal-associated species, *Penicillium verrucosum*, has been shown to produce mycotoxins as well (Krogh et al., 1970).

One of the first products, representing a new generation of more sustainable plant-strengthening products, is JumpStart® (Novozymes, Denmark). This product is an inoculant that contains *P. bilaiae* to increase the availability and accessibility of soil P to plant roots through the colonization of the root system and production of organic acid exudates. It stimulates vigorous root growth, shoot development, increases draught tolerance, and improves yield potential. Furthermore, higher seedling vigor is known to give increased tolerance to pests and diseases, an additional indirect effect of the use of such a “BioAg” product. The BioAg term covers a group of new bio-based products, derived from microorganisms, microbial metabolites or enzymes or that are produced by converting side-streams and residues from biorefineries into higher-value products (Lange et al., 2021; Novozymes, Denmark). It is to be expected that several more species of *Penicillium* that do not produce toxic metabolites could be developed into other types of BioAg products. In 2020, Hansen et al. showed that seed inoculation with *P. bilaiae* and *Bacillus simplex* increased the P concentration in root biomass, along with magnesium, manganese and sulfur, in shoot biomass in low-P soil. These results indicate that the use of the studied microbial inoculants consortium has the potential to improve the nutritional status of winter wheat in low-P soil. It is important to note that the inoculation of seeds with a combination of two or more microorganisms may be more effective than just using one microorganism (Hansen et al., 2020). Developing new BioAg products for more purposes and uses goes hand in hand with finding sustainable, bio-based substitutes for agrochemical fertilizers and pesticides.

**Figure 3:**
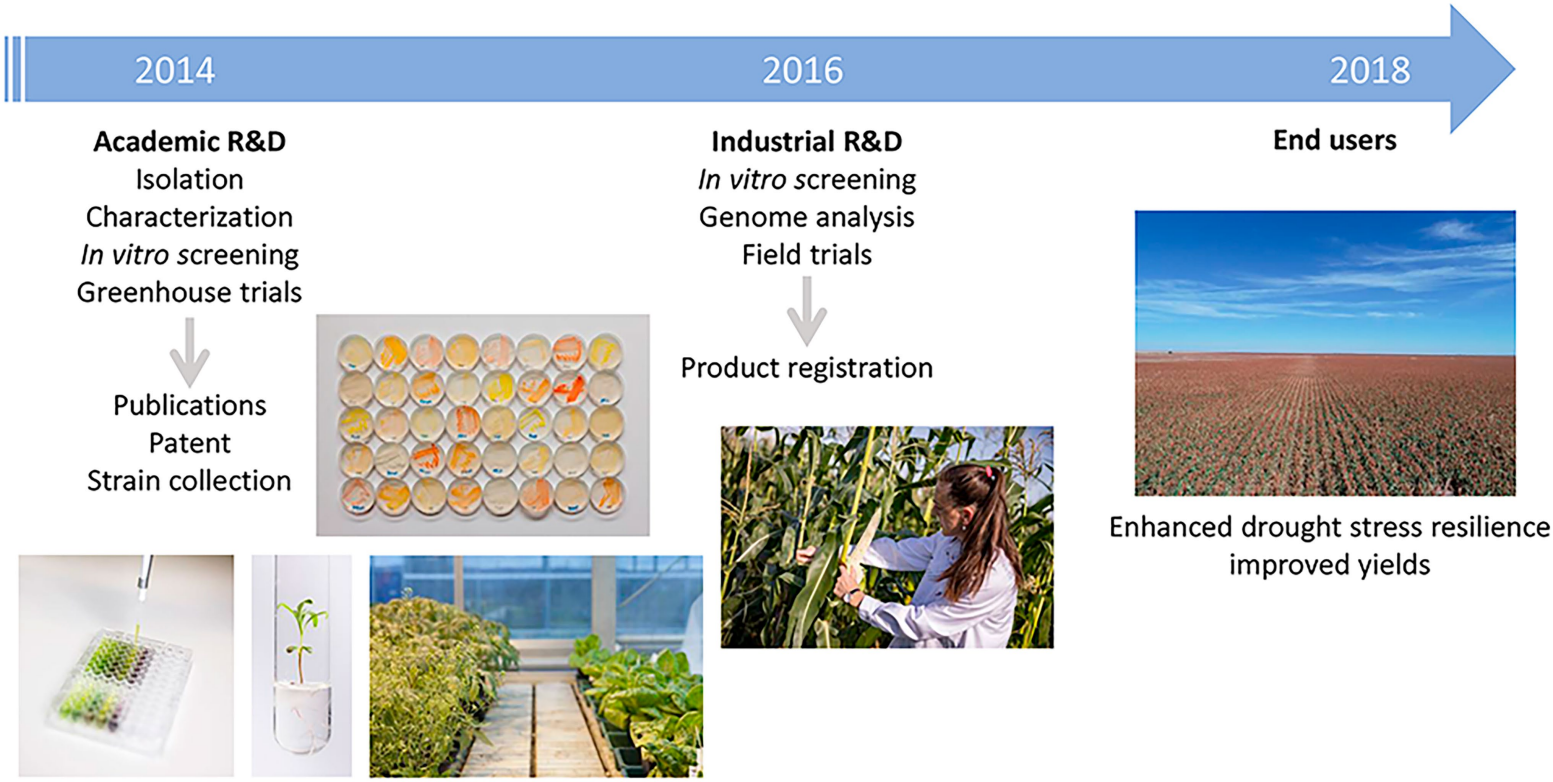
Flowchart describing the development of the products for the improvement of drought-stress tolerance based on the microbial strain. The activities and main outputs of key academic and industrial partners are shown.

Other microbial endophytes, isolated in the process of doing academic research, have led to commercially available products for the improvement of drought-stress tolerance in crop plants. An example is reported in the next success story (case study 4; Table 1; Figure 3). Drought stress is the most important global challenge for crop production with respect to global warming. It is expected that the severity and frequency of droughts will increase in the future and will cause serious plant growth problems for more than 50% of arable land by 2050 (Vinocur and Altman, 2005). In addition to classical breeding and genetic engineering for improved drought stress tolerance in crop plants, there is an increasing interest in the use of PGP microorganisms to secure crop yields under drought stress conditions (Naylor and Coleman-Derr, 2018; Ullah et al., 2019). In 2014, the startup INDIGO Ag (US) was seeking microbial endophytes with the potential to improve plant growth and initiated a collaboration with the Austrian Institute of Technology GmbH (AIT, Austria). In the context of various plant microbiome studies, AIT researchers were routinely isolating plant-associated microorganisms, which were then tested for different traits beneficial to plants, such as PGP or biocontrol. By doing this work, they established a collection of several hundred bacterial isolates from different plant species and compartments. Ten bacterial strains isolated from maize roots or seeds were selected based on indole-3-acetic acid (IAA) production and 1-aminociclopropane-1-carboxylase (ACC) deaminase activity and were rigorously tested for their biochemical properties. Furthermore, the selected strains were assessed in greenhouse trials for their effect on maize seedling growth (Figure 3; Naveed et al., 2014; Mitter et al., 2016). Based on these results, the startup obtained a license for the strains and a patent was filed (US9364005B2, Mitter et al., 2016). Using hyperspectral imaging, in depth testing of the interaction between the strains and various crops was performed, and one *B. simplex* (*Bacillus* sp. S4) strain repeatedly showed efficacy in improving seedling vigor under water and N deficit in winter wheat as well as reproducible PGP in corn under N stress. In parallel, genome analysis revealed a range of genes important for plant-association and potential PGP in the *B. simplex* strain, such as the pathway for the synthesis of the siderophore aerobactin or methylthiolated cytokinins. Following the *in vitro* analysis, the *B. simplex* strain was tested extensively in field trials over 3 years and proved efficiency to enhance drought stress resilience in crops by improving root architecture (Figure 3). In 2018, INDIGO Ag launched a product named Indigo™ 30 containing the strain as flowable powder for the treatment of corn seed in the United States. Today, the strain is available under the product name biotrinsic™ W10 in US, Brazil, Argentina and several EU countries and used for corn, wheat and sorghum.

It is expected that the upcoming generation of PGP-based products will focus on (1) the optimization and development of methods for delivering microbes; (2) the use of co-adjuvants or other molecules that are synergistic with the effects of bacterial or fungal inoculation; and (3) the creation of products containing a consortia of microbes capable of delivering multiple beneficial traits to plants. It has been recently shown in several studies that an improved rhizosphere microbiome can provide disease suppression and strengthen plant growth; however, only a small fraction of the available knowledge has been translated into the field. Therefore, additional research into the dynamic interactions between crop plants, the rhizosphere microbiome, and the environment are necessary to better guide the harnessing of the microbiome to increase crop yield, quality, and sustainability.

### Feed Products and Livestock Health Sector

Omics-based technological advances have also revolutionized our understanding of host-associated microbial communities in livestock health. Animal microbiome research often focuses on the microbial populations that inhabit the gastrointestinal tract (GIT) of animals, in order to determine their role in the animal’s growth, health and disease (Maynard et al., 2012; Van Borm et al., 2015; Bahrndorff et al., 2016; Celi et al., 2017; Peixoto et al., 2021). Functional insights into the animal’s GIT microbiome will lead to advances in animal disease prevention and nutrition through the targeted administration of probiotics, prebiotics, feed additives and customized diets (de Lange et al., 2010; Kiarie et al., 2013; Dawood et al., 2018; Quijada et al., 2020a). Effective functionality of the GIT and its health are important factors in determining animal performance (e.g., body weight gain and milk, meat, and egg production and quality; Celi et al., 2017). The nutritional quality of feed is a critical factor for animals as it has a significant impact on their health, productivity, protection against pathogens and toxins, and the regulation of their immune system (Chaucheyras-Durand and Durand, 2010). Manipulation of the microbiome also has a broad range of applications: from improving nutrition to the protection of the animals against pathogens and toxic compounds. Antimicrobials have been widely used in animals for preventing and treating GIT infections, with the inadvertent consequence of increasing the risk of spreading antimicrobial resistant mechanisms in bacteria (McEwen and Collignon, 2018). Therefore, in 2006, the use of antimicrobials in feeds was banned in the EU and several other countries (Directive 2003/1831/EC, 2003). Several alternatives such as probiotics, prebiotics, enzymes, and phytogenic compounds were utilized to prevent pathogenic bacterial growth and to promote the beneficial GIT microbiota toward improving the feed conversion ratio (Moretti et al., 2018). In principle, probiotics are an effective method to enhance the beneficial GIT microbiome by stabilizing epithelial barriers prone to foodborne pathogens (Deng et al., 2020). Within the last decade, many studies have provided evidence for specific and significant changes caused by microbiome-based feeding that leverages well-described types of feed additives (de Lange et al., 2010; Markowiak and Śliżewska, 2018).

Globally, feed production uses approximately 75% of all arable land. Further, responsible industrial animal production is struggling to solve issues regarding animal welfare, diseases and zoonosis, and find means to cut down the use of antimicrobials and reduce GHG emissions. Thus, it is essential to identify sustainably resourced feed materials (e.g., from food processing side-streams or marine biomass) and select feedstocks with high potential to provide the basis for stabilizing and sustaining a healthy GIT microbiome (Grela et al., 2019; Tomaszewska et al., 2019).

### Microbially Enriched Feed Additives

The fermentation process has been used to improve the nutritional quality and stability of various foods (Aktaş and Akın, 2020). Microbial fermentation can increase the digestibility of most proteins and convert indigestible sugars to lactic acid and prebiotic oligo-elements (Dotsenko et al., 2018). Fermentation of feed not only preserves high-quality feedstuffs for long-term use, but also degrades toxins and anti-nutritional factors and reduces harmful microorganisms in low-quality ingredients (Dai et al., 2020). The feed fermentation process has also been used in preparing livestock liquid diets (Canibe and Jensen, 2012; Missotten et al., 2015). In recent years, attention has been drawn to the use of fermented feedstuffs in dry feed (Navarro et al., 2017).

**Figure 4:**
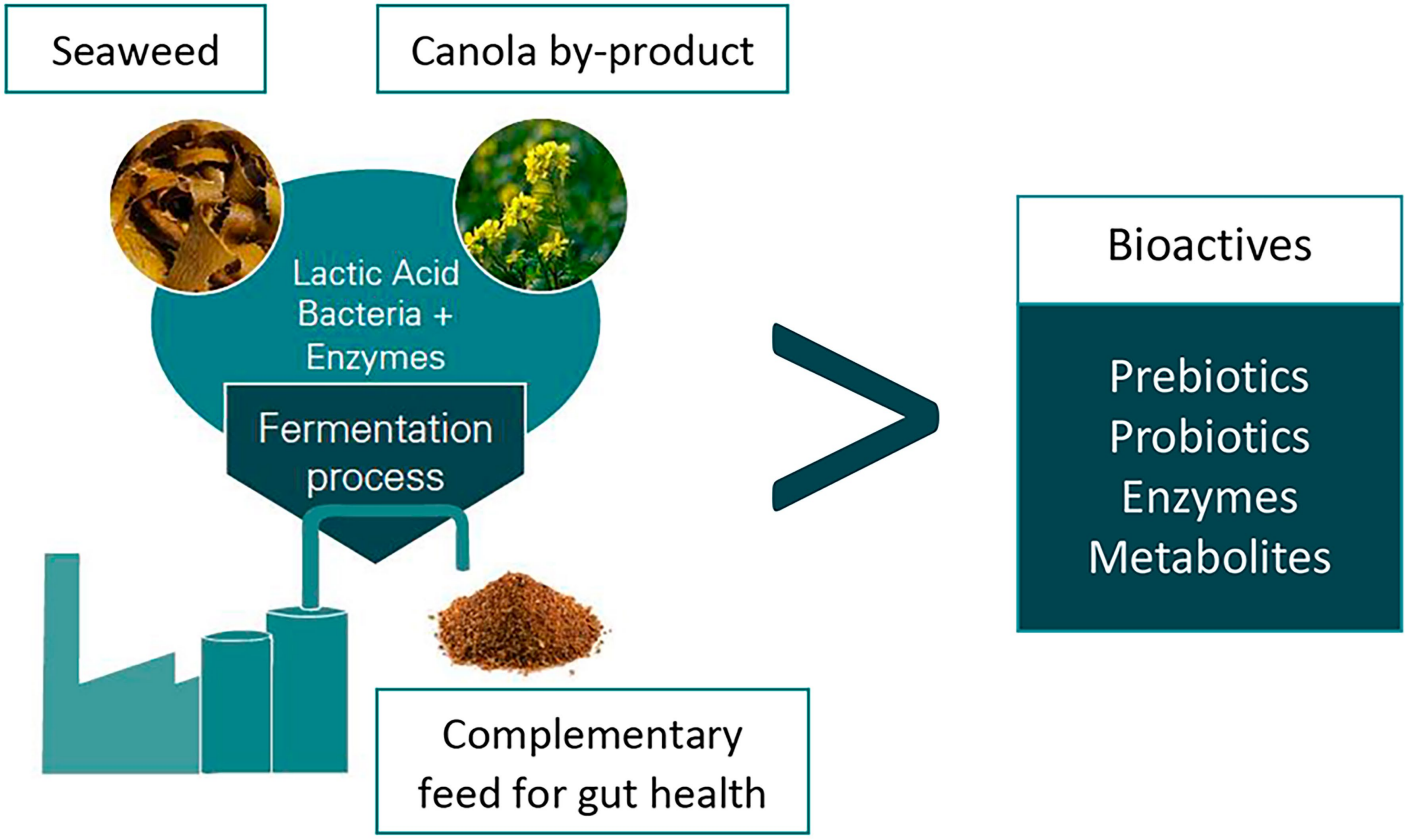
Flowchart for production of the new feed additive EP199. Rapeseed meal and brown seaweed biomass are co-fermented by lactic acid bacteria (LAB).

The weaning of piglets in modern pig production is generally done at an early age and is associated with stresses due to major changes in diet, environment, and social groups. Consequently, weaned piglets experience a reduced feed intake, intestinal and immune dysfunction, as well as increased risk of infection with enteric pathogens (Pluske et al., 1997; Lallès et al., 2004). Thus, the production efficiency of a swine facility is eventually reduced due to stunted growth, post-weaning diarrhoea, and increased mortality of the piglets (Campbell et al., 2013). There is an urgent need to find suitable strategies to enhance the performance and GIT health of weaned piglets, thus substituting the use of antibiotics or veterinary doses of zinc oxide (ZnO). Notably, high dose of ZnO in animal feed, added to prevent weaning-related loss in productivity, must be phased out by 2022 in the EU (European Medicines Agency, 2017). In case study 5 (Table 1), we present a newly developed dry feed additive based on co-fermented rapeseed meal and seaweed (from sustainably sourced feed materials) with lactic acid bacteria (LAB), which promote intestinal development and gut epithelial barrier functions in weaner piglets (Satessa et al., 2020a,b). Piglet performance, intestinal development, and other health indicators were sustained or numerically improved when in-feed ZnO was replaced by this new feed additive (EP100i, European Protein, Denmark). In two separate studies with newly weaned piglets, this dry feed additive enhanced small intestinal villi and colon mucosal development, brush border integrity and reduced signs of small and large intestinal inflammation, and the latter was also observed when the rapeseed meal had been co-fermented with the brown algae *Ascophyllum nodossum* (EP199, European Protein, Denmark; Figure 4). The morphological changes observed in the small intestine increased the absorptive capacity of the gut. This could explain, why performance was sustained or even improved in the piglets fed the pre-fermented rapeseed product in the two studies (Satessa et al., 2020a,b). The solid-state fermentation process, in which the commercial rapeseed product is produced, is a controlled, anaerobic process using cultures of three fermentative LAB (Satessa et al., 2020b). Hence, piglets were provided with not only probiotic bacteria added during the pre-fermentation process, but also with health-promoting bioactive metabolites generated during the pre-fermentation process (Shi et al., 2015; Pessione and Cirrincione, 2016; Drażbo et al., 2018). This increased the microbial biodiversity and robustness of the microbial community in the hindgut (Satessa et al., 2020a). It is furthermore likely that the microbial pre-fermentation process has improved the nutritional value of the rapeseed meal itself, since fermentation in other studies was shown to improve digestibility and neutralize anti-nutritional factors (e.g., tannins, glucosinolates, and phytic acid; Shi et al., 2015; Drażbo et al., 2018). The sustainable development of this product is not only based on the use of sustainably sourced feed material but also on using solid-state fermentation instead of liquid-fermentation, thereby lowering water consumption and energy. Such microbially enriched fermentatively processed feed products are already commercialized as pig-feed additives and contribute to enabling a more sustainable meat production. This microbiome-based feed product developed by the EU feed industry can hence decrease economic losses during production, even when the use of growth promoters are phased out. Indeed, the most important contribution is the reduction of the use of ZnO and antibiotics.

### Probiotic and Prebiotic Feed Supplements

Applications of additives like prebiotics in both animal feed and human food have paved the way for development in the synthesis of oligosaccharide prebiotics (Dotsenko et al., 2018; Mano et al., 2018). Oligosaccharides derived from many natural polysaccharides have been shown to have prebiotic effects (e.g., fructooligosaccharides, galactooligosaccharides, maltooligosaccharides, and gentiooligosaccharides; Al-Sheraji et al., 2013). Xylooligosaccharides (XOS) have recently been suggested as a promising alternative to fructooligosaccharides prebiotics (Aachary and Prapulla, 2011). XOS are sugar oligomers composed of xylose units, which appear naturally in bamboo shoots, fruits, vegetables, milk and honey. Their production at an industrial scale is carried out from lignocellulosic materials (Vázquez et al., 2000). The production of XOS from agricultural residues means that the raw material is cheap and abundantly available. In case study 6 (Table 1) the XOS ability to modify pig gut microbiome was investigated (Dotsenko et al., 2018). XOS were obtained from monocotyledonous biomass, wheat straw, and ryegrass by treatment with different xylanases. The effect of dimers, trimers, tetramers, pentamers, etc. on the pig gut microbiota was evaluated using *in vitro* fermentation of pig fecal samples. The analysis was made from porcine gut samples, isolated from dissected, slaughtered animals. The shifts in the microbial communities were successfully measured by using culture-based methods. Significant differences in the prebiotic potential (suppression of the potential pathogen *Clostridium perfringens* and stimulation of beneficial LAB) were found between non-treated and XOS-treated samples. Differences were also found between the lengths of XOS. These results showed an improvement in porcine gut health after such prebiotic oligosaccharide treatments (Dotsenko et al., 2018). Furthermore, it is possible to produce such prebiotic feed additives affordably and at large scale as an integrated product from a Green Biorefinery that converts biomass to higher value products (Corona et al., 2018; Dotsenko et al., 2018; Parajuli et al., 2018).

Poultry eggs and meat form a major portion of the non-vegan diet and are a major source of protein all around the world, with the highest consumption in developing countries (Stanley et al., 2014). Yet, poultry products are one of the significant sources of foodborne pathogens responsible for high morbidity and mortality globally (Mahami et al., 2019). Another major problem faced by the poultry farmers is the contamination of poultry feed by poisonous compounds caused by mycotoxin-producing fungi. Mycotoxicosis has a drastic impact in poultry animals and includes the impairment of the immune system and translocation of pathogens to other organs, representing a threat to the public health (Galarza-Seeber et al., 2016; Ruhnau et al., 2020). Aflatoxins from *Aspergillus* species are the major contaminant in poultry feed that leads to many of the pathologic effects on poultry, including death (Azeem et al., 2019). Several studies have pointed to the profound benefits of probiotics in managing mycotoxicosis in poultry animals (Moretti et al., 2018; Azeem et al., 2019; Liew et al., 2019; Chang et al., 2020). Moretti et al. (2018) demonstrated the utilization of a probiotic-zeolite (mycotoxin-binding agent) combination to diminish the concentration of aflatoxin B1. *Lactiplantibacillus plantarum* CIDCA 83114 was freeze dried, combined with zeolite, and was incorporated in the feed along with thyme. In the study carried out by Azeem et al. (2019), a combination of six probiotic strains, including *Limosilactobacillus fermentum* FYP 38, *Lactobacillus gallinarum* PL 149, *L. gallinarum* PL 53, *L. gallinarum* PDP 10, *Lacticaseibacillus paracasei* PL 120, and *Limosilactobacillus reuteri* PDP 24 showed anti-aflatoxigenic effects against *Aspergillus flavus* in poultry feed. In case study 7 (Table 1), Liew et al. (2019) reported that aflatoxin B1 induced an altered gut microbiome in rats, while the probiotic *Lacticaseibacillus casei* Shirota helps to restore the gut microbiome. The addition of probiotic strains belonging to the species *Bacillus subtilis*, *L. casei* and *Candida utilis* to the broiler diet supported a stable gut microbiota, the degradation of mycotoxins and reduction of toxicity, the improved histological lesions, and growth performance (Chang et al., 2020).

Among the poultry-borne pathogens, *Salmonella* and *Campylobacter* are the most important affecting the humans (Heredia and García, 2018). Control strategies such as probiotics prevent GIT colonization by *Campylobacter* by various mechanisms of action that include competitive exclusion, antagonism, and immunomodulation (Deng et al., 2020). Bacterial probiotic strains belonging to species and genera *Bacillus subtilis*, *Bifidobacterium* spp., *Enterococcus* spp., and *Lactobacillus* spp. have been selected for modulation of the poultry gut microbiota and to prevent pathogens (Rubio, 2019). Yeast supplementation as a feed additive in poultry farming is also receiving attention because it contains intracellular components such as amino acids, enzymes, cofactors, peptides, carbohydrates, salt components, MSG (monosodium glutamate) RNA, in addition to extracellular cell wall components such as glycoproteins, β-glucans and mannooligosaccharides and thus is a rich source of protein, fiber, and minerals that has resulted in increased host growth and improved health (Shurson, 2018; Hameed et al., 2019). The *in vivo* evaluation of β-Glucan isolated from yeast showed anti-inflammatory, and immune modulatory effects (Bacha et al., 2017). Commercially available products such as PoultryStar® (Biomin, Austria) or Probiotics Daily (Durvet, United States) have multiple strains of LAB, whereas other products contain *B. subtilis* and yeast strains in addition to LAB, e.g., FloraZone (Refit animal care, India) or Lavipan® (JHJ, Poland).

Microbiome modulation in the GIT is an innovative and emerging approach to control farm animal disease and foodborne pathogens. A customized combination of microbial-based therapies could promote animal health and contribute to the practice of sustainable husbandry.

### Food Production Sector

Microbiome-based innovations have the potential to improve food processing and human health. The microbiological analysis of foods continues to be heavily reliant on the use of classical culture-based approaches involving agar plates and incubators. While these have value, they are laborious, time-consuming and require the selection of specific growth media based on the microorganisms predicted to be present, or cause for concern, with respect to that specific food (Cocolin et al., 2013; Cocolin and Ercolini, 2015; De Filippis et al., 2018). This means that problems caused by microorganisms that are non-cultivable and/or not expected to be present in the food will be difficult to detect. Extending the knowledge of the cultivable and non-cultivable food microbiota is pivotal to unravel its potential role in the sustainability, safety, and quality of food production. In this regard, novel HTS approaches offer a wide range of exciting new possibilities for food research, as already discussed (Ercolini, 2013; Ferrocino and Cocolin, 2017; Walsh et al., 2017; De Filippis et al., 2018; Quijada et al., 2020b; Ferrocino et al., 2022). Bokulich and Mills (2013) described that the development of a microbial community within a food production environment is driven by community-wide adaptations in response to substrates or conditions within each environmental niche. The recent application of HTS techniques has expanded our knowledge of microbial communities in foods and their processing environment with an unprecedented level of detail (Doyle et al., 2017; McHugh et al., 2020; Cobo-Díaz et al., 2021).

### The Role of the Microbiome in Food Fermentation

The meat microbiome has an important role in the conversion of the components of meat into several metabolites, with a consequential major impact on physical and organoleptic properties, and the quality and safety of the final products (Cobo-Díaz et al., 2021; Franciosa et al., 2021). The initial microbiota is influenced by several factors such as the season, slaughter procedure, transport conditions, manufacturing equipment, factory environment and operators (Franciosa et al., 2021). Re-contamination is often a stochastic process throughout the slaughter line, which is complex to monitor (Mann et al., 2016). Multi-step monitoring is required to identify microbial shifts, obtain useful information on fermentation dynamics, and evaluate possible health risks (Mrkonjic Fuka et al., 2020).

Mediterranean countries produce numerous Protected Geographical Indication (PGI), Protected Designation of Origin (PDO), or Traditional Specialty Guaranteed (TSG) fermented sausages (Aquilanti et al., 2007; Franciosa et al., 2018). Traditional fermented sausages are considered a significant part of the Mediterranean cuisine and represent a gastronomical heritage with significant regional and international value. The different products develop a microbiota that is specific to the region where they are produced and that are responsible for the fermentation and quality of the products. Therefore, in order to protect the traditional process used for making the sausages, it is essential to understand the microbial dynamics during fermentation and to select autochthonous microorganism as potential starter cultures (Franciosa et al., 2018). Industrialized production often involves the addition of starter cultures to standardize the process. The quality of the final product is dependent, among other factors, on the initial microbiome composition of the raw materials and the subsequent modification thereof along the process chain, which can drastically affect its function. In recent decades, several culture-dependent and -independent studies were carried out to describe the evolution of the microbiota in fermented meat and meat products (Mauriello et al., 2004; Cocolin et al., 2009; Kesmen et al., 2012; Quijada et al., 2018).

Case study 8 (Table 1) relates to fermented sausages, where a multi-omics approach was used to reveal the importance of the autochthonous microbiome in precision meat fermentation (Ferrocino et al., 2018; Franciosa et al., 2021). The use of meta-omics approaches, coupling DNA sequencing with metabolomics, was used to connect sensorial quality of the final products with the metagenomic repertoire of sausages obtained both without starters or using with a commercial starter culture (a mixture of *Lactilactobacillus sakei* and *Staphylococcus xylosus* strains; Ferrocino et al., 2018). The presence of the starter culture, in particular *L. sakei*, ensured rapid microbial growth, a high acidification rate and fast consumption of fermentable substrates. A decline in the relative abundance of *Enterobacteriaceae* was observed, as well as in the microbial diversity. Metagenomic analysis of the sausages made with the starter showed higher level of acidity and the resulting taste was characterized as pungent, vinegary, cheesy, and weedy. On the other hand, the indigenous microbiome from spontaneous fermentation (composed by *Lactobacillus delbrueckii subsp. lactis*, *L. sakei*, *Leuconostoc citreum*, *Leuconostoc gelidum* and *S. xylosus*) displayed higher counts of genes involved in fatty acid biosynthesis and amino acid metabolism. From the metabolome dataset, higher amounts of medium- and long-chain fatty esters enhanced the sensory profile of these sausages conferring the sausages a “fruity wine, waxy sweet apricot, and banana brandy” flavor. As a result, consumers preferred the spontaneous fermented sausages due to their flavor and odor characteristics. In contrast, products made with the starter culture that boosted the production of acetic acid were found to be unacceptable by the consumers (Ferrocino et al., 2018). Autochthonous microbiomes display a huge pool of genes with adapted metabolic functions that can potentially provide beneficial effects in terms of sensorial properties, but they may also add to the variability of the fermentation process. Meat is an ecological niche for *L. sakei* and the study from Franciosa et al. (2021) showed that a different starting microbiome influenced the fermentation process *via* various metabolic pathways. The different metabolomic characteristics of the batches used in this study were not only linked to the species level, but also to intra-species strain-level biodiversity. A culturomics approach was then used to isolate and characterize the indigenous microbiome to develop an autochthonous microbiome starter culture with the ability to drive the fermentation process and display optimal final sensorial and aromatic characteristics in the final product, as assessed by a test for consumer preference. Therefore, data generation through multi-omics approaches is enabling us to study the microbiome at the highest resolution and to discover the potential of an autochthonous microbiome as a starter culture. This precision fermentation strategy ensures high quality final products, while simultaneously meeting the consumers’ preferences regarding organoleptic characteristics. Another advantage of a multi-omics approach is the ability to detect biomarkers related to the safety of the products (such as genes underlying antimicrobial resistance, biogenic amines, or other virulence factors; Lopez et al., 2020).

In addition to meat products, the microbiota of fermented dairy products have also been widely studied due to its importance in the manufacturing process. Kefir is a functional, viscous, and slightly carbonated fermented milk having origins in the Caucasian, Tibetan, and Mongolian mountains and being associated with a wide range of health benefits (Kabak and Dobson, 2011). LAB, acetic acid bacteria, and yeasts constitute its complex microbial community and unique ecosystem. For example, yeasts stimulate LAB growth by producing B-group vitamins and hydrolyzing milk proteins, while LAB ferment lactose and decrease the pH (Álvarez-Martín et al., 2008). Kefir has been manufactured artisanally for centuries by using characteristically elastic, slimy, white to yellow and irregular “grains.” Kefir grains have a strange cauliflower-like structure of different sizes that comprises coagulated milk proteins and mucous polysaccharides known as kefiran (Dobson et al., 2011; Kabak and Dobson, 2011; Leite et al., 2013). Additionally, kefir can be produced industrially using LAB and yeasts as starter cultures. Different methods, including PCR-denaturing gradient gel electrophoresis (PCR-DGGE) and HTS, have been used to study of the complex microbial composition of kefir grains and drinks and have uncovered the influence of the geographical origin, milk source and manufacturing method on the kefir microbiome (Kesmen and Kacmaz, 2011; Leite et al., 2012, 2013; Marsh et al., 2013; Diosma et al., 2014; Garofalo et al., 2015; Dertli and Con, 2017). Case study 9 (Table 1) describes the microbiota of four home-made kefir samples (both grains and drinks) from different geographical regions in Greece and four industrial kefir drinks. To do this, classical microbiological analysis was coupled with molecular techniques and taxonomic marker gene amplicon HTS (Kazou et al., 2021).

The results showed that *Lactobacillus* was the most abundant bacterial genus in the grains, while *Lactobacillus* and *Lactococcus* were the most abundant in home-made kefir drinks. The bacterial microbiota of industrial kefir drinks differed to the grains and home-made kefir samples, as they were mainly dominated by common genera used as starter or adjunct cultures in the dairy industry. More specifically, the genera *Streptococcus*, *Lactococcus*, *Lactobacillus*, *Lacticaseibacillus*, and *Bifidobacterium* were predominant with varying abundances among the industrial samples. The differences among the samples were more evident when considering the fungal microbiota. Indeed, *Saccharomyces* dominated in grains and home-made kefir drinks. Industrial kefir drinks showed a more diverse fungal microbiota where the yeast *Kluyveromyce*s was the most abundant genus, although its relative abundance varied depending on the sample (Kazou et al., 2021). The results ultimately showed that certain bacterial and fungal genera were mainly associated with either the home-made or the industrial kefir samples. Evaluation of the Greek kefir microbiota can be used to support the verification of its safety and to find correlations with the product’s technological, sensorial, and functional properties (health benefits). This study, which constitute the first report on the kefir microbiota in Greece combining classical microbiological and amplicon-based metagenomics analyses, attracted significant interest from industry with interest in harnessing the knowledge to produce new functional dairy products.

It should also be noted that an in-depth study of the technological potential of the isolates should be performed, since strains belonging to the same species/genus can harbor a different genes and thus exert a different impact on the ecosystem. Additionally, a single strain is not able to confer specific characteristics to the product itself in most cases, but rather in combination with other strains with a cocktail of different genetic repertoires. Understanding these interactions is the new frontier of the food-omics approach. Moreover, the implementation of modeling based on meta-omics data can help prevent yield loss during food production and, at the same time, enable the prediction of product characteristics. This implementation requires the integration of data and bioinformatic tools into a predictive model based on the starting microbiome, and some limitations still need to be overcome, such as access to genomic and metabolomic databases specific for microbial groups, (e.g., LAB).

### Food Processing Environment-Associated Microbes

Current estimations of global food loss and waste lie at around 1.3 billion tons every year (Vilariño et al., 2017). This corresponds to approximately one-third of the food produced globally, resulting in an immense waste of resources, and contributes significantly to global GHG emissions. On average, about one-third of the global food loss and waste is attributed to the retail and consumer level, while 24%–30% is lost during production, 20% is lost post-harvest, and the rest is lost in the field prior to harvest (Kummu et al., 2012). While the main reason for food waste at the consumer level is over-shopping, the reasons for food loss can be diverse, including spoilage, defects, malfunction, or overproduction (Ishangulyyev et al., 2019).

Cheese “pinking” is a discoloration defect that affects a range of different ripened cheese varieties (Daly et al., 2012). Despite being a subject of investigation over the last century, the basis for this phenomenon has been the subject of much debate (Daly et al., 2012). The defect does not have an impact on flavor or safety, but it does have significant economic consequences as the appearance of the pink color on the surface or within the cheese can result in products not being released to, or being withdrawn from, the market. Classical microbiology had failed to identify differences in the microbial composition of this pink discoloration defect in standard cheeses (Daly et al., 2012). Thus, a culture-independent DNA sequencing approach, using both 16S rRNA gene and shotgun metagenomic sequencing, was employed in the next success story (case study 10; Table 1; Quigley et al., 2016; Yeluri Jonnala et al., 2021). This demonstrated a higher relative abundance of microorganisms from the genus *Thermus* in defect cheeses. These microorganisms had not previously been found in cheese, most likely due to difficulties in cultivating them. The role for *Thermus* in the pinking defect phenomenon was subsequently confirmed when a strain of *Thermus thermophilus* was spiked into cheese and “recreated” the pinking defect (Quigley et al., 2016). Armed with the knowledge that *Thermus* can be a cause of the discoloration defect, it was then possible to develop a qPCR-based assay to identify the route *via* which these microbes entered the food chain (Quigley et al., 2016) and, in turn, develop strategies to prevent their entry and/or control them (Yeluri Jonnala et al., 2021). The identification of *Thermus* as a contributor to the cheese-pinking defect was notable for providing one of the first examples of the merits of employing HTS-based approaches to identify the microbial basis for a defect with an unknown source along the food chain. The identification of the causative agent then provided cheesemakers with a means of identifying its source and employing approaches to control it. Although *Thermus* has been shown to contribute to the cheese pinking phenomenon, there may be other microbes and/or factors involved in other specific cases of cheese pinking. Notably, different microbes have been associated with other discoloration defects in cheese, such as a purple rind discoloration of surface-ripened cheeses (Kamelamela et al., 2018).

Microbial food spoilage is responsible for a considerable amount of food waste and can cause foodborne diseases in humans. Indeed, the food industry faces major and continuing challenges in trying to lower the extent to which food products become contaminated with pathogenic or spoilage bacteria during primary processing. This is especially true for animal-derived products like poultry, eggs, milk, and pork, which are the main vehicles for food-borne diseases (European Food Safety Authority and European Centre for Disease Prevention and Control, 2018). Therefore, preventing microbial food spoilage is a major concern for health authorities, regulators, consumers, and the food industry. However, the contamination of food products is difficult to control because there are several potential points during production, processing, storage, distribution, and consumption, where microorganisms come in contact with the product. The environmental microbiota from processing plants have often been addressed as sources of microbes that potentially affect the quality attributes of meat. The microbiota involved in meat food-processing steps are often found on processing surfaces or tools, highlighting the importance of hygienic practices in influencing the food microbiota (De Filippis et al., 2013; Hultman et al., 2015). HTS revealed that undesirable microorganisms can be found everywhere in the food processing environment and easily contaminate food (De Filippis et al., 2013; Mann et al., 2016).

**Figure 5:**
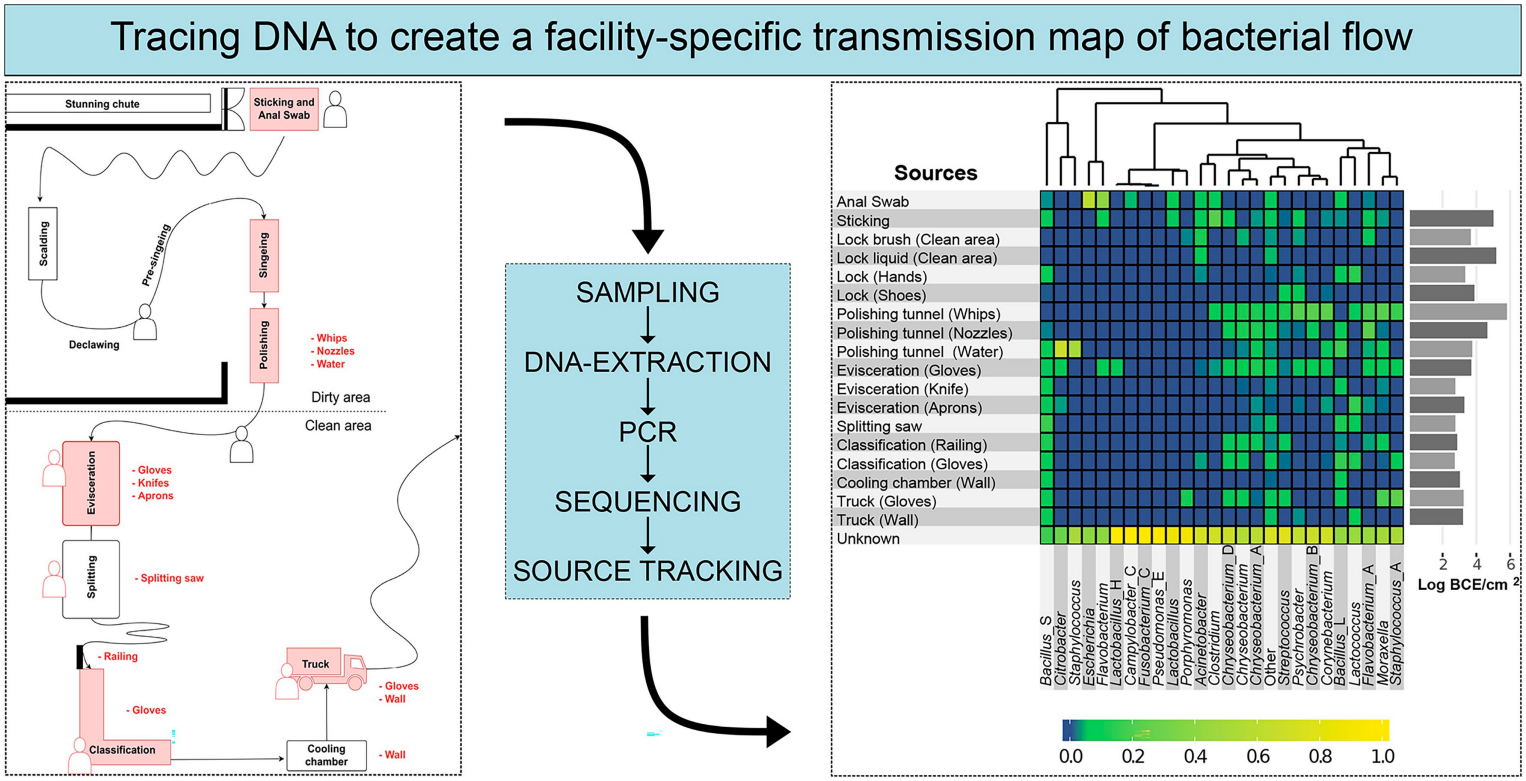
Schematic workflow describing microbial source tracking in slaughter facilities based on 16S rRNA gene amplicon HTS. The left box illustrates a schematic map of a meat processing plant with sampling areas marked in red. The right box shows the final result, a heatmap with the predicted relative contribution of specific genera from the sampled source environments. The figure was modified from Zwirzitz et al. (2020) published under http://creativecommons.org/licenses/by/4.0/.

Full-length 16S rRNA gene amplicon HTS was used in case study 11 (Table 1) to provide insights into bacterial community structure throughout a pork-processing plant (Figure 5; Zwirzitz et al., 2020). Specifically, the proportion of bacteria on meat that is initially not animal-associated and is therefore transferred during cutting *via* personnel, equipment, machines, or the slaughter environment was investigated. The obtained data were used to create a facility-specific transmission map of bacterial flow, which predicts previously unknown sources of bacterial contamination. This map enabled the linking of specific bacterial groups to environmental sources and provided the facility with essential information for targeted disinfection and the establishment of a renovation plan to eliminate explicit contamination sources. For example, it became apparent that *Staphylococcus* originated from the water in the polishing tunnel and *Escherichia* from anal swab samples taken when pigs entered the facility. Several other prominent meat spoilage microorganisms were most likely transferred from the gloves of employees, a railing at the classification step, and the whips in the polishing tunnel (Figure 5; Zwirzitz et al., 2020). This knowledge led to novel disinfection interventions that improved the hygiene status in the facility. Each species had a unique transmission profile, demonstrating that not all species were distributed throughout the whole facility but rather that they occupied particular environmental niches from which they were disseminated. Therefore, a high taxonomic resolution is necessary for microbial source tracking in food processing plants. While whole genome sequencing of isolates is still necessary for molecular epidemiology purposes, full-length 16S rRNA gene amplicon HTS could be a cost-effective solution for regular monitoring and the identification of general bacterial transmission routes in food processing facilities. HTS techniques can also be extended to other food-processing environments to gain knowledge about microbial transmission routes, improve hygiene standards, increase food safety and minimize food waste in general.

These studies provide a nice example to show that HTS of food and food chain samples is not a purely academic exercise but rather can have a meaningful impact on industry. Simplifying and standardizing protocols, from sample collection to bioinformatic analysis, will eventually allow for the regular use of HTS in food production facilities and ultimately result in a better understanding of microbial contamination along the food supply chain.

### Human Health Sector

The microbiome and its implications for human health have attracted significant interest in recent decades. Sequencing the human gut microbiome in health and disease states (Yatsunenko et al., 2012) promptly led to efforts to find out how such microbiomes could be stabilized, restored, or modified to favor holobiont symbiosis. Many gut microbial species and/or their produced metabolites have been shown to contribute to host immunity, nutrient processing, and energy harvesting and were further linked to the prevention or management of multiple diseases (Dahiya et al., 2017; Fung et al., 2017). A dysbiosis of our microbiome is often associated with disease. Driving factors for the structure and function of our microbiome are individual genetics, gender, age, and lifestyle factors. Diet is further considered a main factor (Singh et al., 2017). In contrast to Western diet, which is associated with a number of inflammatory, chronic and allergic gut diseases (Statovci et al., 2017; Zinöcker and Lindseth, 2018), other diets, e.g., the Mediterranean diet, which is rich in fiber, and traditional Asian diets, which include many fermented foods, are thought to support gut health (Şanlier et al., 2017; Tosti et al., 2018; respectively). Emerging evidence also indicates that the microbiome composition of individuals might help to predict the risk of developing chronic diseases (Rampelli et al., 2018) and responses to dietary interventions (Zeevi et al., 2015; Berry et al., 2020). However, understanding of the microbiome’s role in dietary-mediated health effects is still too limited to provide solid recommendations. Dietary guidelines by regulatory bodies scarcely address the role of the microbiome in human nutrition (Sanz et al., 2018). Continuing to generate evidence on the role played by specific gut microbes through interactions between diet and our health is essential to advance toward robust dietary recommendations. It is crucial to understand the key role of nutrition and the human microbiome in personalizing health and disease management as well as to align regulatory and policy actions that facilitate translation to society.

### Health Promoting Metabolites from Fermented Food

Fermented foods are defined as “foods made through desired microbial growth and enzymatic conversions of food components” (Marco et al., 2021) and have been part of our daily diet for more than 14,000 years. In the past, food fermentation was mainly introduced to avoid food spoilage. Much later, it was also recognized that fermented foods can have beneficial health effects. In the former section, we highlighted the pivotal role of the food microbiota in determining the quality of food products. Moreover, many bacteria and fungi have been isolated from fermented foods and considered as probiotics due to their beneficial properties for human health (Parvez et al., 2006; Marco et al., 2017, 2021). Nevertheless, how certain microorganisms drive food fermentation, are transferred across the food production chain, persist in the final product and, potentially, colonize the human gut is poorly understood. This is also true for various health promoting agents (living bacteria, enzymes, prebiotics, etc.). More work here would help us to (a) drive the process of food fermentation in a more targeted way and (b) use probiotics or their metabolites for new forms of therapy diseases based on gut dysbiosis, including both infectious and non-communicable diseases, such as bowel inflammatory disorders, cardiovascular disease, allergies, or cancer.

Bronchial asthma is one of the diseases that has become very important worldwide in recent years. In case study 12 (Table 1), we illustrate how a specific metabolite (D-tryptophan) obtained from food fermenting bacteria has immunomodulatory potential and can mitigate this disease. Toward doing so, Kepert et al. (2017) used a number of strains belonging to the genera *Bifidobacterium*, *Lactobacillus*, *Lacticaseibacillus* and *Lactococcus*, among others. These were obtained from fermented food and their immunomodulatory activity was evaluated. Cell-free supernatants derived from *Lactococcus* sp. strains were shown to decrease the secretion of CCL17 by a human Hodgkin lymphoma T-cell line (KM-H2) without affecting cell viability. *L. casei* W56 was then used for the enrichment and stepwise chemical characterization of candidate metabolites, revealing tryptophan as the responsible compound for the immunomodulatory effect. Enantiomeric separation of the purified subfraction confirmed the presence of D- and L-tryptophan, whereas the corresponding growth medium used as a control contained only L-tryptophan. Oral supplementation of D-tryptophan in mice with induced asthma (by Ovalbumin) increased D-tryptophan serum levels significantly, indicating enteric uptake and systemic distribution. Pretreatment of mice with D-tryptophan for 3 days and throughout experimental asthma induced significant shifts in the gut microbiome of the treated mice resulting in an increase of overall diversity. At the same time, supplementation improved airway hyperreactivity in response to methacholine. Allergic airway inflammation reduced gut microbial diversity, but when fed to mice, D-tryptophan increased gut microbial diversity and ameliorated the disease, demonstrating the importance of the gut-lung axis in health (Kepert et al., 2017). These findings support the concept that defined bacterial products can be exploited as novel preventative strategies for chronic immune diseases. D-tryptophan could be considered either a drug, which should be used to treat patients with asthma or a food additive, which could protect the gut microbiome from a dysbiosis despite a Western diet and other lifestyle factors which have negative impact on the gut microbiome. The next steps will strongly depend on the intended application, either as a drug or food additive, as regulatory authorities treat these very differently.

### Targeting Gut Microbiome to Improve Human Health

Obesity has reached epidemic proportions, which is mainly a consequence of unhealthy dietary habits that are based on foods rich in fats and simple sugars, in addition to a sedentary lifestyle. Obesity severely affects the health status of our society and leads to an increase in health expenditure. Indeed, the Organization for Economic Co-operation and Development (OECD) has estimated that the absence of effective preventive measures for obesity will severely increase the percentage of new cases of diabetes (60%), cardiovascular diseases (18%), dementia (11%), and cancers (8%) between 2020 and 2050, with the consequent increase of premature deaths and the reduction of life expectancy (OECD, 2019). The interactions of the gut microbiota with dietary compounds and the host seem to determine our vulnerability to obesity and its complications (Sonnenburg and Bäckhed, 2016). For example, excessive intake of high-fat foods and simple sugars induces immune dysregulation in the gut, partly triggered by diet-induced microbiota changes (Winer et al., 2016). Dietary fibers are recognized worldwide for their beneficial effects on host health and well-being (Makki et al., 2018). They include resistant starch and non-starch polysaccharides often found in vegetables including legumes, and cereals. Such polysaccharides are known to serve as substrates for the gut microbiota. Besides the well-known claim of improved digestive health, new evidence has emerged regarding the role of these polysaccharides and the microbiome in preventing metabolic disorders, increasing calcium absorption and bone health, boosting immunity and restoring eubiosis (Whisner et al., 2016; He et al., 2017; Beukema et al., 2020; Li et al., 2021; Song and Song, 2021). However, meeting the nutritional recommendations of 30 g/day seems challenging.

In case study 13 (Table 1), a public-private partnership between the National Research Institute for Agriculture, Food and the Environment (INRAE, France) and a bakery (Bridor, France) has aimed to provide more fiber with their product Amibiote® (meaning biota friend), a new commercialized bread (Ranaivo et al., 2022). The recipe includes 7 fibers rigorously selected based on the beneficial effects documented by the INRAE, while the company performed the technological development to provide a multi-fiber bread product that was non-distinguishable by eye from the control bread. The consumption of the multi-fiber bread doubled the daily fiber intake in comparison to the control bread and contributed to promoting gut microbiota diversity and functions. Besides the nutritional qualities of this multi-fiber bread, it also showed ability to manage hypercholesterolemia and improve insulin sensitivity (Ranaivo et al., 2022). This multi-fiber bread increased the *Parabacteroides distasonis* population, a gut bacteria documented for its anti-inflammatory and anti-obesity properties (Cuffaro et al., 2020; Ezeji et al., 2021).

**Figure 6:**
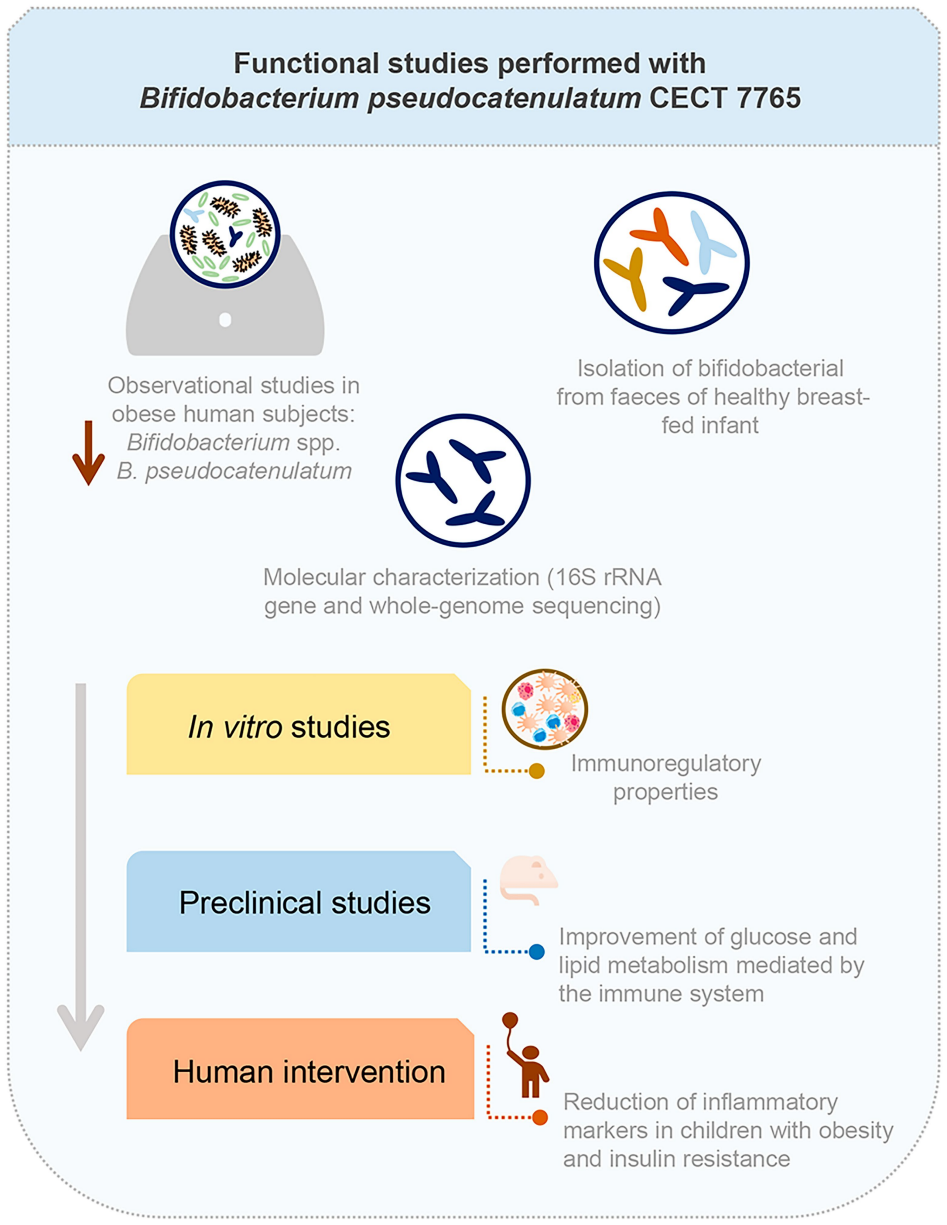
Schematic representation of the process followed to identify and assess the probiotic potential of *Bifidobacterium pseudocatenulatum* CECT 77.

In the final success story (case study 14; Table 1), a probiotic strain, *Bifidobacterium pseudocatenulatum* CECT 7765, was developed for mitigating metabolic complications associated with obesity in humans (Cano et al., 2013; Moya-Pérez et al., 2014, 2015; Benítez-Páez et al., 2016; Sanchis-Chordà et al., 2019). Previous descriptive studies had shown associations between *Bifidobacterium* species, such as *B. pseudocatenulatum*, and a metabolically healthy phenotype (Salazar et al., 2015; Zhang C. et al., 2015). In light of this evidence, a collection of bifidobacterial strains isolated from healthy breast-fed babies were screened for their immunoregulatory properties in macrophages using *in vitro* cultures, which are responsible for driving obesity-associated inflammation that causes insulin resistance and chronic disorders. Based on these results, the strain *B. pseudocatenulatum* CECT 7765 was selected for further trials. The strain was first identified at species and strain level, as required by regulatory bodies, by 16S rRNA gene sequencing and further whole genome sequencing (Figure 6; Benítez-Páez et al., 2016). Then, the effects and mode of action of *B. pseudocatenulatum* CECT 7765 in an animal model of diet induced obesity were evaluated in pre-clinical trials. In these trials, it was demonstrated that the administration of the strain to obese mice reduced body weight gain and serum lipids, and improved oral glucose tolerance and insulin sensitivity (Figure 6; Cano et al., 2013). The effect of *B. pseudocatenulatum* CECT 7765 on gene and protein expression in the liver showed that this strain had modified the expression of key regulators of fatty acid and cholesterol metabolism and transport, and lipid and glucose levels in the liver, which supports the beneficial metabolic effects of this bacterial strain in the obesity model (Moya-Pérez et al., 2014). These effects were partly mediated through the restoration of the intestinal and peripheral immune homeostasis (Moya-Pérez et al., 2015). Briefly, *B. pseudocatenulatum* CECT 7765 reduced obesity-associated systemic inflammation by restoring the balance between regulatory T cells and B lymphocytes and reducing pro-inflammatory cytokines of adaptive and innate immunity in different tissues and endotoxemia (Moya-Pérez et al., 2015). In 2019, Sanchis-Chordà et al. proved the efficacy of *B. pseudocatenulatum* CECT 7765 together with dietary counselling in obese children with insulin resistance in a placebo-controlled intervention trial lasting 13 weeks (Figure 6). In the group receiving the probiotic strain, there was a significant decrease in circulating high-sensitive C-reactive protein and monocyte chemoattractant protein-1 and an increase in high-density lipoprotein cholesterol and omentin-1, compared to the control group. The beneficial effects of the intervention on inflammatory markers and lipid profiles indicated that *B. pseudocatenulatum* CECT 7765 intake together with dietary recommendations could improve the inflammatory status of children with obesity and insulin resistance, reducing the risk of developing chronic disease in the future (Sanchis-Chordà et al., 2019).

Body-weight regulation is not entirely under voluntary control and biological, genetic, and environmental factors critically contribute to obesity (Sanz et al., 2018). In a condition with such a complex etiology, the administration of a probiotic bacterium (*B. pseudocatenulatum* CECT 7765) cannot solve the problem alone but, together with dietary counselling, could help to reduce inflammation and the risk of developing chronic metabolic complications more effectively than dietary changes alone (as shown in the clinical trial; Sanchis-Chordà et al., 2019). The case presented here provides an example of all the steps necessary in the development of a probiotic product to bring it to the market. The *B. pseudocatenulatum* CECT 7765 strain and its use for obesity was patented (WO2012/076739A1; Sanz et al., 2012) and the patent was licensed to a company, which is scaling-up the production of the strain to be introduced in a product to aid in the control of obesity in humans.

The generation of robust scientific evidence on the effects of specific intestinal microorganisms or products thereof on health-outcomes and their mode of action is needed to facilitate legal approval under different regulatory frameworks. For the approval of microorganisms as foods supplements bearing health claims, the scientific criteria applied by the European Food Safety Authority (EFSA) during the evaluation process should be considered at the very beginning of the research program (publicly available EFSA Guidance documents on health claims published in the EFSA Journal). For approval of a microorganism to be used as a drug, the regulatory framework is not as well established, but both the Food and Drug Administration (FDA) and the European Directorate for the Quality of Medicines and Health Care (EDQM) codified these as live biotherapeutic products. The approval of these innovative products is promising but also constitute a challenge due to the limited experience of both applicants and regulators in defining quality and safety criteria and developing suitable study designs to demonstrate efficacy. Despite this, collaboration between the Pharmabiotic Research Institute (PRI) and industrial partners and scientists provides a roadmap (Paquet et al., 2021).

## Conclusion and Future Perspectives

Agrifood companies recognize the potential in understanding the microbiome and translating this knowledge into products. Interested parties range from food ingredient producers, consumer goods companies and medical device companies to biotech firms and technology service providers. The food industry is further preparing to develop personalized diets and specific foods for particular target groups in order to prevent or treat certain chronic conditions. Meanwhile, industrial and academic partners are calling for public–private partnerships to stimulate the translation of scientific knowledge into new products and treatments.

Microbiome research has benefited greatly from the improvement in different multi-omics technologies, such as DNA HTS (Thomas and Segata, 2019). HTS of amplicons of taxonomic maker genes, such as the 16S rRNA gene, 18S rRNA gene or ITS regions, constitutes a quick and cost-effective approach to characterize microbial communities. However, the resolution of this technique is not sufficient to resolve closely-related species and is affected by the reference database, the length and type of amplicon that is sequenced, the operon copy number and the intra-species sequence variability. These hurdles could be overcome by using a shotgun metagenomics approach, where the total DNA extracted from a sample is directly subjected to DNA sequencing. This approach allows for a higher resolution investigation of microbial communities occurring in a sample (including viruses, prokaryotes and eukaryotes) as well as their genomic potential. However, shotgun metagenomics requires greater sequencing depths, increasing the cost, and demands additional computational resources compared to gene amplicon HTS. Moreover, in most cases the results from microbiome studies remain descriptive. Therefore, the use of DNA sequencing should be combined with other omics-approaches, such as RNA sequencing (meta/transcriptomics), (meta) proteomics and metabolomics, to unravel the entire picture of the microbiome and their theatre of activity. The research approaches applied in the case studies discussed here demonstrate that microbiome investigation is improved when analyses complementary to HTS are applied. Moreover, cultivation experiments are still highly valuable for deepening insight into the function of distinct microbiota members and still offer the possibility to capture processes that would otherwise be not explainable by meta-omics approaches. Culturomics studies including the ISO reference methods may not be replaced by HTS in the near future but rather complemented. The use of integrative approaches in microbiome studies to decipher preliminary observations is essential to expedite future developments along the “Farm-to-fork” context.

While the relevance of these approaches to food and food chain microbiology is clear, challenges remain with respect to simplifying sequencing and bioinformatics approaches so that they can be employed by non-experts within agrifood companies. Additionally, regulation and standards will need to be put in place to facilitate their application for routine quality and safety testing. Despite this, the all-important first steps have been made to facilitate the more widespread application of HTS as a powerful tool for agrifood chain applications.

To conclude, it is important to remark that the case studies presented here are a subset of examples of potential applications of microbiome-based research in the agrifood field selected within the MicrobiomeSupport consortium members and their partners. The microbiomes of soil, plants and animals are pivotal for ensuring human and environmental health. Research and innovation on microbiomes in the agrifood system are constantly advancing, and a better understanding of these microbiomes will be a key factor in producing highly nutritious, affordable, safe, and sustainable food.

## Author Contributions

RO, SUW, TK, AS, ES, and MW: conceptualization. All authors substantially contributed to collecting and writing the case studies. RO and SUW drafted the manuscript. RO edited and prepared the submitted version of the manuscript. All authors made a substantial, direct, and intellectual contribution to the work, and revised and approved the last version of the manuscript for its publication.

## Funding

This work was supported by the European Union’s Horizon 2020 R&I programme under grant agreement No. 818116 (MicrobiomeSupport).

## Conflict of Interest

At the time of writing, SS was employed by Indigo Ag. RdS and JA was employed by Symbiomics Microbiome Solutions. YS had a patent on a *Bifidobacterium* strain for obesity licensed (WO2012/076739A1). AS and BM had a patent on the plant-endophyte combinations and uses therefor (US9364005B2).

The remaining authors declare that the research was conducted in the absence of any commercial or financial relationships that could be construed as a potential conflict of interest.

The handling editor BM declared a shared affiliation with the authors MO, MR-P, and YS at the time of review.

## Publisher’s Note

All claims expressed in this article are solely those of the authors and do not necessarily represent those of their affiliated organizations, or those of the publisher, the editors and the reviewers. Any product that may be evaluated in this article, or claim that may be made by its manufacturer, is not guaranteed or endorsed by the publisher.

## Footnotes

^1^ https://pubmed.ncbi.nlm.nih.gov/

^2^ https://www.microbiomesupport.eu/

## Abbreviations

BNF: Biological nitrogen fixation
GIT: Gastrointestinal tract
GHG: Greenhouse gases
HTS: High-throughput DNA sequencing
ISO: International Organization for Standardization
LAB: Lactic acid bacteria
PGP: Plant growth-promoting
R&D: Research and development
XOS: Xylooligosaccharides
